# Analysis and Characterization on Dynamic Recrystallization in Casting AZ31 Mg Alloys Under Plane Strain Compression

**DOI:** 10.3390/ma13030522

**Published:** 2020-01-22

**Authors:** Li Xu, Minghua Xiang, Jun Wang, Jun Zhang, Chenning Wang, Chao Xie

**Affiliations:** Faculty of Mechanical Engineering and Mechanics, Ningbo University, Ningbo 315211, China; 176000058@nbu.edu.cn (L.X.); 1711081092@nbu.edu.cn (M.X.); 1811081012@nbu.edu.cn (J.W.); 1611081466@nbu.edu.cn (J.Z.); 176000055@nbu.edu.cn (C.W.)

**Keywords:** Mg alloy, twinning, dynamic recrystallization, strain rate, temperature

## Abstract

Studies on twinning, twin-induced dynamic recrystallization (TDRX), and their temperature and strain rate dependences are of considerable significance to the ultimate strength and plastic formability of the coarse-grained Mg alloys during severe plastic deformation. Plane strain compression tests were conducted on the parallelepiped samples of casting AZ31 Mg alloys. The twinning and recrystallization behaviors close to and away from the crack boundaries were characterized using electron backscatter diffraction. The results show: (1) with increasing strain rate for tests, the extension twin proliferates significantly. Due to the local stress concentration, the TDRX is more active in the area close to the crack tip and exhibits the positive strain-rate sensitivity as twinning; (2) the TDRX is not only stress-favored but also closely links to the temperature. However, the TDRX is not utterly proportional to the temperature. Compared to 400 °C, 300 °C is more beneficial to the TDRX, achieving the higher strength and plastic deformability. The main reason is that the higher strain-hardening rate and flow stress at the higher strain rate and lower temperature motivates the transformation from twinning to the fine twin-walled grains more efficiently, and the stress-favored TDRX is crucial to refine grains and continue plastic deformation for the casting Mg alloys with coarse grains.

## 1. Introduction

Mg alloys present a great industrial application prospect because of their low density and high specific strength. However, the hexagonal close-packed structure inevitably causes notably insufficient formability. Recently, a typical severe plastic deformation process, the high-strain-rate rolling [1,2,3,4] that can significantly refine grains and enhance the mechanical performances of Mg alloys, has drawn widespread attention from industrial and scientific fields. However, due to the insufficient slip systems and the strong basal texture, cracks are still frequently formed at the edges and surfaces of Mg alloy samples during the high-strain-rate rolling. It was experimentally found that the finished product rate strongly depends on the strain-rate and temperature effects on the microstructural evolution [3,4,5,6,7,8,9,10,11,12], which urgently needs to be identified.

The impact tests on the AZ31 Mg alloy plates along the normal direction [13] indicated that the increased strain rate results in increased flow stress, and that the stress-strain curves show positive strain-rate sensitivity because the strain-rate sensitive double twinning with the high critical resolved shear stress predominantly accommodates the plastic strain. In addition, Zhang et al. [14] found that the higher strain rate can more efficiently active the twin-induced dynamic recrystallization (TDRX) [15], and the failure strain of the casting AZ31 Mg alloys tested by the plane strain compression (PSC) is enhanced by the TDRX-induced softening at the higher strain rate.

In addition to the strain rate, the temperature effect on the TDRX is also significant. In Mg and its alloys, the TDRX exists at an extensive temperature range, including low (below 250 °C) [16], intermediate (250–350 °C) [5,16,17,18], and high temperature (above 350 °C) [19,20,21] ranges. In the temperature range of 150–300 °C, the volume fraction of TDRX in pure Mg escalates with increasing temperature [16]. However, in the temperature range of 300–400 °C, the TDRX fraction gradually drops down with increasing temperature. Generally speaking, a higher temperature is more beneficial to dynamic recrystallizations (DRXs). However, for the TDRX, the temperature effect is non-monotonous.

The purposes of this study are to elucidate how the test strain rate and temperature influence the twinning and TDRX and to understand the twinning and TDRX effects on the strength and hot formability of casting Mg alloys with coarse grains.

## 2. Materials and Methods

### 2.1. Plane Strain Compression

**Figure 1:**
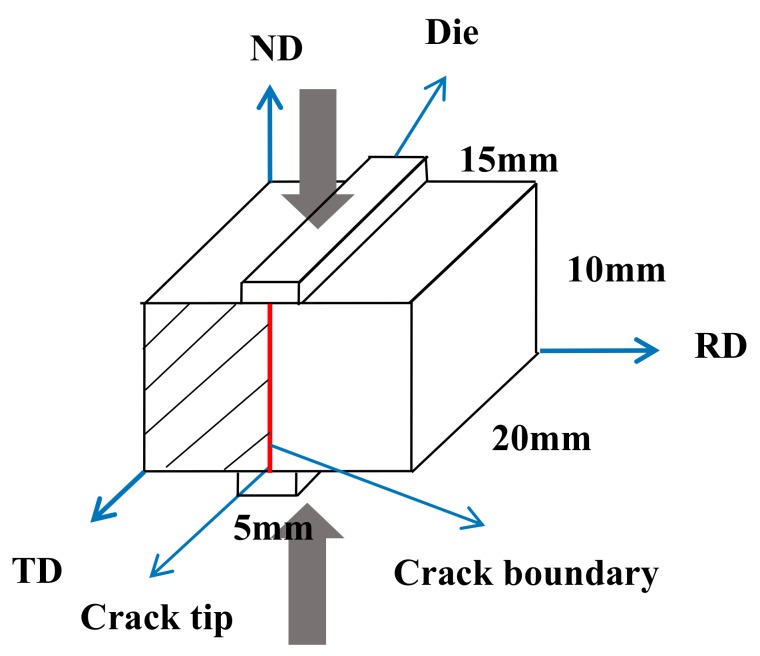
Schematics of PSC and EBSD characterization plane (shaded area).

Because the stress, strain, and heat flow of the PSC are similar to those of the rolling process [22,23], PSC is employed to quantitatively simulate the high-strain-rate rolling. The parallelepiped samples used for PSC tests, with the initial dimensions of 20 mm in width, 15 mm in length, and 10 mm in thickness, were prepared by the wire-electrode cutting on the casting AZ31 Mg alloy ingot that was made in the Key Laboratory of High Temperature Wear Resistant Materials Preparation Technology of Hunan Province, University of Science and Technology, Xiangtan, China (Table 1 shows the element content of AZ31 Mg alloy). The PSC tests were carried out under vacuum using the Gleeble-3500 thermo-mechanical simulator (Dynamic Systems Inc., New York, NY USA). The Graphite lubricants and tantalum foils were used to eliminate friction between samples and the tools. The samples were heated to the temperature of 300 °C by a thermocouple and then held for 10 min prior to the compression tests. The compression geometry [24] and the Electron Backscatter Diffraction (EBSD, AZtec Nordlys Max data acquisition system, Oxford Instruments, Abingdon, UK) characterization plane is shown in Figure 1. The initial dimensions of the parallelepiped samples are 15 × 20 × 10 mm, and the width of the die is 5 mm. The PSC tests were conducted on the samples until fracture, the fractured samples with small dimensions and crack boundaries were directly processed as the EBSD samples, and no additional cutting was needed. It is worth noting that, the ultimate strain rate of the simulator is 160 s^−1^, the high-strain-rate rolling usually be carried out at the average strain rate of above 10 s^−1^, and therefore three different typical strain rates of 10 s^−1^, 80 s^−1^, and 160 s^−1^ were selected for the PSC tests.

### 2.2. Electron Backscatter Diffraction

**Figure 2:**
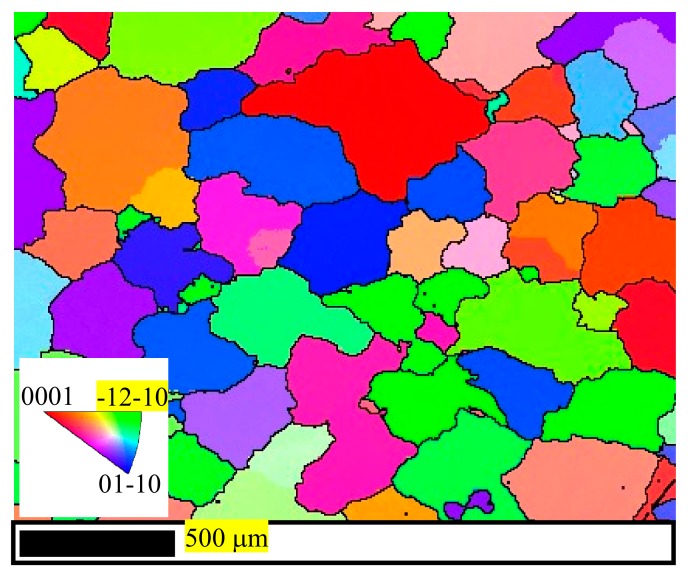
An EBSD inverse pole figure-Z map of initial texture.

The EBSD samples were the transverse direction (TD) plane containing the crack boundaries. These samples were first ground using SiC papers and then polished with diamond paste with the sequence of W3.5, W2.5, W1, W0.5, and W0.25. After being polished, the samples were etched using a mixture solution containing 10 mL HNO_3_, 30 mL acetic acid, 40 mL H_2_O and 120 mL alcohol. Finally, the EBSD data were measured by the SU5000 Hitachi scanning electronic microscopy (SEM) (Hitachi High-Technologies GLOBAL, Tokyo, Japan) equipped with the Oxford Aztec Nordlys Max data acquisition system, and the microstructures and texture were analyzed using the HKL Channel 5 system (5.11.20405.0, Oxford Instruments, Abingdon, UK). The SEM and EBSD operation voltage, spot intensity, magnification, and step size are 20 KV, 70 pA, 500, and 0.5 μm, respectively. The inverse pole figure along the compression direction (Z direction) of the initial texture before deformation is shown in Figure 2. It is obviously shown that all grains are of random orientation with an average grain diameter of 200.96 μm, and no evidently strengthened texture exists in the casting alloys. The image quality (IQ) maps shown in following figures are the figures distinguishing recrystallizations, substructures, and deformed materials. Using HKL software, it is set that the red regions represent the fully recrystallized materials, the silver regions represent the substructured materials, and the aqua zones represent the deformed materials. The minimum grain boundary misorientation angles to separate subgrains and grains are 2° and 15°, respectively. In addition, the yellow, green, and blue lines represent the extension twinning (ETW), contraction twinning (CTW), and double twinning (DTW) boundaries, respectively.

## 3. Results

### 3.1. Stress–strain Relationships

**Figure 3:**
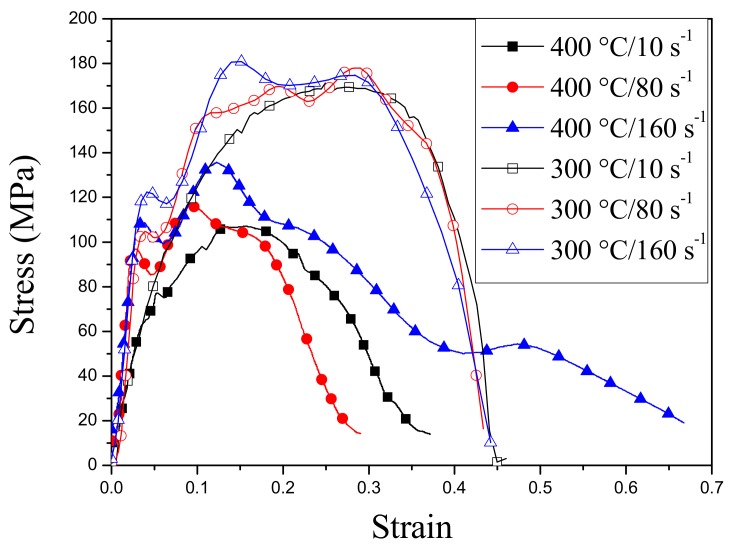
True stress–strain relationships of the PSC samples tested at three different strain rates (10 s^−1^, 80 s^−1^, and 160 s^−1^) and two different temperatures (300 and 400 °C [14]).

The true stress–strain curves of the casting AZ31 Mg alloys tested by the PSC at three different strain rates of 10 s^−1^, 80 s^−1^, and 160 s^−1^ and two different temperatures of 300 and 400 °C [14] are shown in Figure 3. It is obviously shown that, at the same strain rate, both the samples tested at 300 and 400 °C almost keep the same hardening rate at the early hardening stage (stain: 0–0.025). However, at the latter hardening stage, the samples tested at 300 °C present clearly higher hardening rates, which significantly increase the flow stress and ultimate strength. At 300 °C and 160 s^−1^, the flow stress and ultimate strength are the greatest. Furthermore, compared with the samples tested at 400 °C, the samples tested at 300 °C present the longer segments of the approximately ideal plastic deformation, and the sample tested at 160 s^−1^ achieves the longest one, within which the true stress approximately remains unchanged, and the true strain increases greatly. Basically, at 300 °C, the coarse-grained AZ31 Mg alloy samples have the better ultimate strength and plastic deformability than those at 400 °C. In addition, the double-peak phenomenon evidently exists in the stress–strain curves with a higher strain rate. Generally, a single peak is understood as the transition of the twinning-induced hardening and the recrystallization-induced softening within coarse-grained Mg alloys [15]. With increasing strain rate, twins become more active within the recrystallized grains, and the secondary hardening and softening could happen and lead to the secondary peak.

### 3.2. Twinning and Recrystallizations

**Figure 4:**
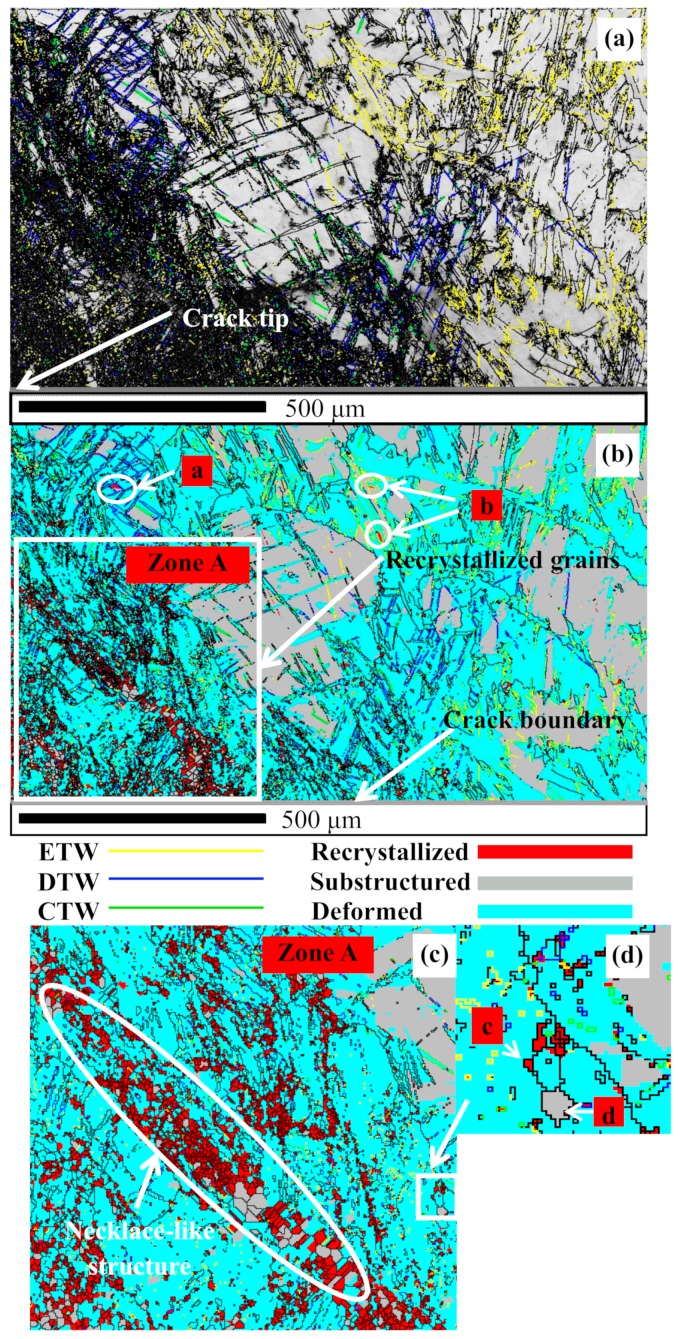
IQ maps of the area containing the crack (300 °C, 10 s^−1^): (**a**) twinning and fine grain distribution; (**b**) twin-induced dynamic recrystallization (TDRX); (**c**) necklace-like structure; (**d**) grain boundary bulging.

**Figure 5:**
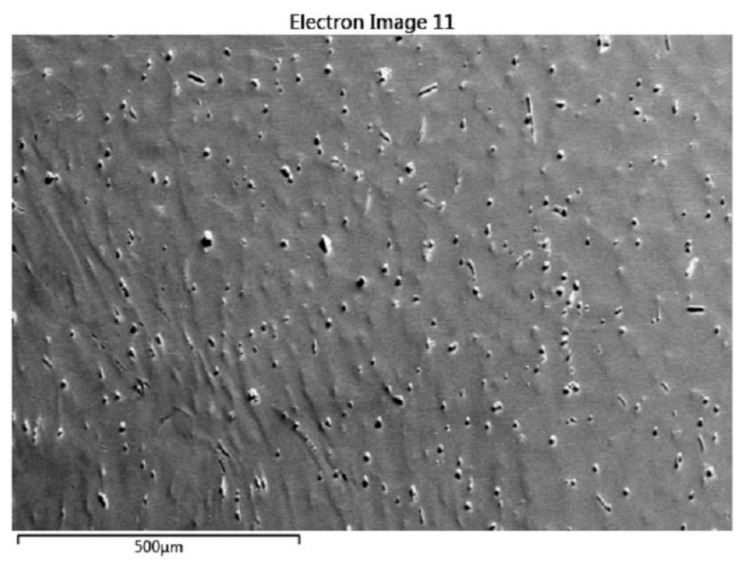
A SE image of the region of Figure 4a.

As shown in Figure 4a that, at the 300 °C and 10 s^−1^, a massive amount of fine grains emerges within the area close to the crack tip, and twinning is also very profuse (the statistical fraction is shown in Table 2). The black regions are actually the grain boundaries with a high density. Because the recrystallized grains are fine and dense within the area close to the crack tip, the numerous grain boundaries set as black lines look like black regions. Within the area far away from the crack tip, a majority of twinning has not evolved into the DRX, and only a tiny minority of ETW and DTW has evolved into the DRX that inherits the c-axis orientation of parent twinning (as shown in Figure 4b, the grain marked by a is a DTW-induced DRX grain, and the grains marked by b are ETW-induced DRX grains). Within the area close to the crack tip, the profuse TDRX has been formed into the necklace-like structure composed by the small grains with smooth grain boundaries (Figure 4c). Only very few grains are evolved from the grain boundary bulging (the grains marked by c and d in Figure 4d). For the whole plane of Figure 4a, the misorientation angle distribution shows that, at the strain rate of 10 s^−1^, the misorientation angles of grain boundaries are more evenly distributed, and relatively less twinning boundaries are detected, which should be attributed to the reorientation of the TDRX grains. Figure 5 is the second-electronic (SE) image of the region of Figure 4a.

**Figure 6:**
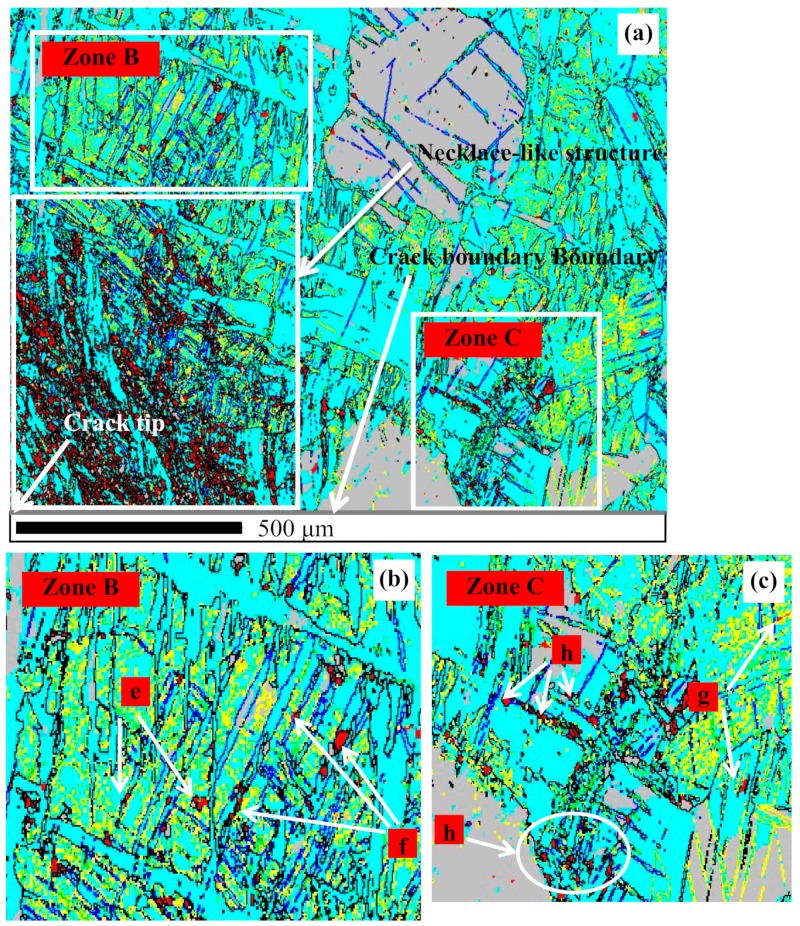
IQ maps of the area containing the crack (300 °C, 80 s^−1^): (**a**) Twinning and necklace-like structure; (**b**) ETW-induced and DTW-induced DRX within the area far away from the crack tip and grain boundary; (**c**) ETW-induced and DTW-induced DRX within the area far away from the crack tip but close to the crack boundary.

Figure 6a shows that the profuse ETW and DTW grow at 300 °C and 80 s^−1^. Near the crack tip, the active twinning benefits slip, accommodates plastic deformations, accumulates a high density of dislocations and the strain energy within itself, and finally drives the TDRX and the formation of the necklace-like structure with smooth boundaries (the volume fraction of the TDRX is clearly more than that at 10 s^−1^). Due to the higher stress and strain within the area close to the crack tip, the continuous reorientation gradually diverts the TDRX grain boundaries from the twin boundaries. However, within the areas (Figure 6b,c) far away from the crack tip, the new grains marked by e and g have just been evolved from the ETW, the new grains f and h have just been evolved from the DTW, the grains inherit the orientation of parent twinning, and the necklace-like structure has not been formed. For the whole plane of Figure 6a, the misorientation angles are evenly distributed at the large grain boundary range, except for the angle of 86.3°.

**Figure 7:**
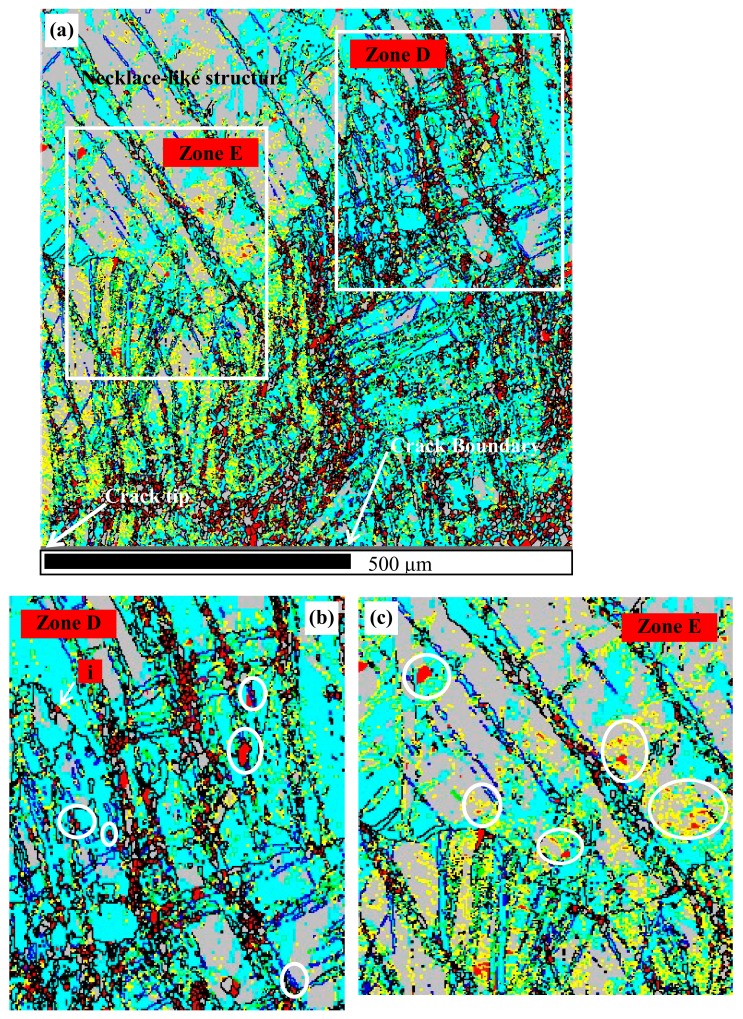
IQ maps of the area containing the crack (300 °C, 160 s^−1^): (**a**) Twinning and necklace-like structure; (**b**) DTW-induced DRX; (**c**) ETW-induced DRX.

**Figure 8:**
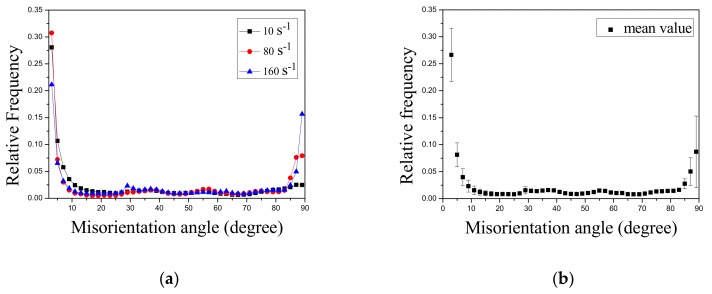
(**a**) Misorientation angle distribution and (**b**) error bar of the AZ31 samples containing the crack tested at 300 °C.

Figure 7a shows that, at 300 °C and 160 s^−1^, the higher strain rate results in the formation of a massive amount of ETW in the whole area, particularly in the area close to the crack tip. In addition, the ETW-induced recrystallization (Zone E shown as Figure 7c), DTW-induced recrystallization (Zone D shown as Figure 7b), and the necklace-like structure frequently and extensively emerge within the whole area. There is only rare discontinuous DRX that is based on the grain boundary bulging (the grain marked by i and shown in Figure 7b). It is worth noting that although the TDRX is prevailing, a number of ETW have not yet evolved into new grains, and therefore Figure 8a shows a high peak value at the misorientation angle of 86.3°.

The misorientation angles were detected by EBSD measurement across the whole plane shown as Figure 4aFigure 6a and Figure 7a and calculated by HKL software. The misorientation angle range is 2°–90° (Figure 8a. In total, 1441 grains in Figure 4a, 2144 grains in Figure 6a and and 2309 grains in Figure 7a were considered for the statistical analysis of 10 s^−1^, 80 s^−1^, and 160 s^−1^, respectively. The error bars of the misorientation angle distribution has been added to show the frequency dispersity at different misorientation angles (Figure 8b). Figure 8b shows the frequency dispersity of small-angle grain boundaries is also comparatively larger in addition to that of ETW boundaries.

**Figure 9:**
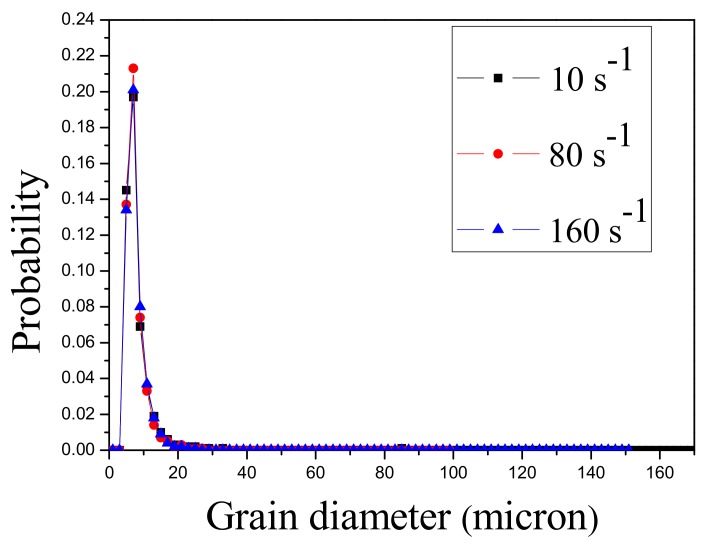
Grain size distribution of AZ31 samples containing the crack tested at 300 °C.

The grain size distribution of AZ31 samples containing the crack tested at 300 °C is shown in Figure 9. There is no great difference among the three strain rates, and the dispersity of the grain sizes is not very high.

Table 2 statistically shows that the volume fraction of the ETW is almost the highest at any temperature and strain rate, owing to the ETW requiring the lowest shear stress. In addition, the volume fraction of the ETW significantly increases with increasing strain rate at 300 °C, which means that the ETW presents the positive strain-rate sensitivity. It is generally believed that twinning is insensitive to temperature; however, at the same strain rate, the fraction of the ETW at the temperature of 300 °C is clearly less than that at 400 °C. This should be ascribed to that more ETW has evolved into the TDRX at the temperature of 300 °C, and the orientation of the new grain boundaries gradually deviates from that of the parent ETW due to reorientation.

Table 3 shows the comparison of the average, the standard deviation, the maximum, and the minimum of grain sizes at different temperatures and strain rates. The grain sizes were estimated by the HKL software. The critical misorientation angle for grain boundaries is set as 15°, the boundary completion is allowed down to 2°, and the twin boundaries with the misorientation angles of 86.3° (ETW), 56.2° (CTW), and 37.5° (DTW) are disregarded. For three different strain rates, the average and standard deviation of grain sizes at 300 °C are much smaller than the counterparts at 400 °C, which means that a majority of the detected grains at 300 °C are the fine DRX grains with close diameters, and the fine grains at 400 °C are not so profuse as those at 300 °C. The higher dispersity at 400 °C indicates that the grain refinement caused by TDRX is completely not uniform and thorough, and fine and coarse grains coexist. Generally, the DRX is driven by both stress and temperature, and the higher the temperature is, the more efficiently the DRX occurs. However, it is experimentally found that the temperature of 300 °C is more beneficial to the TDRX and grain refinement than the temperature of 400 °C because the higher strain-hardening rate and flow stress at 300 °C (see Figure 3) drives the TDRX more efficiently. In addition, it is believed that the stress-favored TDRX can effectively weaken the basal deformation texture, enhance the basal slip activity, and reduce the ETW activity [15]. This is why the samples tested at 300 °C show the longer segments of the approximately ideal plastic deformation (see Figure 3).

## 4. Discussion

The temperature and strain-rate effects on the stress–strain relationships and microstructural behaviors of casting AZ31 Mg alloys during PSC were tested and characterized in this study. The Correlations among the loading conditions, microstructural behaviors, and macroscopic performances should be clarified.

(1) The volume fractions of ETW and TDRX at 300 °C significantly increases with increasing test strain rate, showing the positive strain-rate sensitivity. Generally, twinning is insensitive to the test temperature. However, it is statistically found that the fraction of the ETW at 300 °C is much less than that at 400 °C. Furthermore, the average and standard deviation of grain sizes at 300 °C are much smaller than the counterparts at 400 °C. The above two points are mainly attributed to that more ETW has evolved into the TDRX at 300 °C, more small twin-walled grains with smooth boundaries are formed, and the subsequent reorientation gradually diverts the orientations of new grains from the parent twinning. Macroscopically, the higher strain-hardening rate and flow stress at 300 °C are capable of driving the TDRX and grain refinement more efficiently, causing the abnormal temperature effect. This is different from the general mechanism that the higher the temperature is, the more easily the DRX occurs.

(2) At the same strain rate, the samples tested at the temperatures of 300 °C and 400 °C almost maintain the same hardening rate at the early hardening stage (strain: 0–0.025). However, at the later hardening stage, the strain-hardening rate at 300 °C was obviously higher, and therefore the flow stress and ultimate strength also evidently increase. At the temperatures of 300 °C, the sample tested at 160 s^−1^ has the greatest flow stress and ultimate strength resulting from the strain hardening by twinning. Furthermore, compared with the samples tested at 400 °C, the samples tested at 300 °C present the longer segments of the approximately ideal plastic deformation resulting from the strain-softening by the TDRX. The sample tested at 160 s^−1^ achieves the longest segment of the approximately ideal plastic deformation. During this process, the stress basically remains constant, while the strain increases substantially. Therefore, for the casting AZ31 Mg alloys, the higher strain rate and appropriate temperature, which active the positive strain-rate sensitive twinning to achieve a higher strain-hardening rate and facilitate the stress-favored TDRX to adequately reduce the ETW activity, increase basal slip, and realize strain softening, can achieve the better ultimate strength and plastic deformability during the PSC tests that model the high-strain-rate rolling.

## Figures and Tables

**Table 1:** Element content of AZ31 Mg alloy.

Elements in AZ31 Mg Alloy	Weight Ratio (wt.%)
Mg	95.30%
Al	3%
Zn	1%

**Table 2:** Volume fraction of twinning at different strain rates and temperatures.

Temperature (Unit: °C)	Strain Rate (Unit: s^−1^)	Twinning Volume Fraction (Unit: %)		
		CTW	DTW	ETW
300	10	2.64	5.20	2.79
	80	1.83	4.06	4.89
	160	1.04	2.27	9.12
	Average	1.84	3.84	5.59
400 [14]	10	0.08	0.92	17.80
	80	2.33	3.57	7.78
	160	0.18	0.82	16.30
	Average	0.86	1.77	13.96

**Table 3:** Grain size and ultimate strength at different strain rates and temperatures.

**Temperature (unit: °C)**	300			400 [14]		
**Strain rate (unit: s^−1^)**	10	80	160	10	80	160
**Average grain diameter (unit: μm)**	8.5402	8.0934	8.2257	34.958	33.280	28.486
**Standard deviation of grain diameters (unit: μm)**	7.3771	5.0217	5.3573	52.758	57.305	50.148
**Maximum of grain diameters (unit: μm)**	169.02	99.384	150.3	438	520.5	453
**Minimum of grain diameters (unit: μm)**	5.3524	5.3524	5.3524	1.5	1.5	1.5
**Ultimate strength** **(unit: MPa)**	171.16	177.88	180.78	105.76	115.50	135.30

## References

[1] Sanjari M., Farzadfar S.A., Utsunomiya H., Sakai T., Essadiqi E., Yue S. (2012). High speed rolling of Mg–3Al–1Zn alloy: texture and microstructure analysis. Mater. Sci. Tech.

[2] Chai F., Zhang D.T., Zhang W.W., Li Y.Y. (2014). Microstructure evolution during high strain rate tensile deformation of a fine-grained AZ91 magnesium alloy. Mater. Sci. Eng. A.

[3] Zhu S.Q., Yan H.G., Chen J.H., Wu Y.Z., Liu J.Z., Tian J. (2010). Effect of twinning and dynamic recrystallization on the high strain rate rolling process. Script. Mater..

[4] Zhu S.Q., Yan H.G., Liao X.Z., Moody S.J., Sha G., Wu Y.Z., Ringer S.P. (2015). Mechanisms for enhanced plasticity in magnesium alloys. Acta Mater..

[5] Zhu S.Q., Ringer S.P. (2018). On the role of twinning and stacking faults on the crystal plasticity and grain refinement in magnesium alloys. Acta Mater..

[6] Liu X., Zhu B.W., Xie C., Zhang J., Tang C.P., Chen Y.Q. (2018). Twinning, dynamic recrystallization, and crack in AZ31 magnesium alloy during high strain rate plane strain compression across a wide temperature. Mater. Sci. Eng. A.

[7] Zhu B.W., Liu X., Xie C., Wu Y.Z., Zhang J. (2019). {10–12} extension twin variant selection under a high train rate in AZ31 magnesium alloy during the plane strain compression. Vacuum.

[8] Zhu B.W., Liu X., Xie C., Liu W.H., Tang C.P., Lu L.W. (2018). The flow behavior in as-extruded AZ31 magnesium alloy under impact loading. J. Mag. Alloy..

[9] Zhu S.Q., Yan H.G., Chen J.H., Wu Y.Z., Du Y.G., Liao X.Z. (2013). Fabrication of Mg–Al–Zn–Mn alloy sheets with homogeneous fine-grained structures using high strain-rate rolling in a wide temperature range. Mater. Sci. Eng. A.

[10] Nakano H., Yuasa M., Chino Y. (2014). {10–12} twins in the rolled Mg–Zn–Ca alloy with high formability. J. Mater. Res..

[11] Ayoub G., Rodrigez A.K., Shehadeh M., Kridli G., Young J.P., Zbib H. (2018). Modelling the rate and temperature-dependent behaviour and texture evolution of the Mg AZ31B alloy TRC sheets. Philos. Mag..

[12] Ostapovets A., Bursik J., Krahula K., Kral L., Serra A. (2017). On the relationship between {11–22} and {11–26} conjugate twins and double extension twins in rolled pure Mg. Philos. Mag..

[13] Wan G., Wu B.L., Zhang Y.D., Sha G.Y., Esling C. (2011). Strain-rate sensitive of textured Mg–3.0Al–1.0Zn alloy (AZ31) under impact deformation. Script. Mater..

[14] Zhang J., Xie C., Zhu B.W., Liu X., Wang X.F., Ma T.F., Peng W.F., Shu X.D. (2018). Strain-rate effects on twinning, dynamic recrystallization and their competition of casting AZ31 Mg alloys during the plane strain compression. Mater. Res. Exp..

[15] Xie C., He J.M., Zhu B.W., Liu X., Zhang J., Wang X.F., Shu X.D., Fang Q.H. (2018). Transition of dynamic recrystallization mechanisms of as-cast AZ31 Mg alloys during hot compression. Int. J. Plast..

[16] Sitdikov O., Kaibyshev R. (2001). Dynamic recrystallization in pure magnesium. Mater. Trans..

[17] Wu Y.Z., Yan H.G., Zhu S.Q., Chen J.H., Liu A.M., Liu X.L. (2014). Flow behavior and microstructure of ZK60 magnesium alloy compressed at high strain rate. Trans. Nonferrous Met. Soc. China.

[18] Xu S.W., Kamado S., Matsumoto N., Honma T., Kojima Y. (2009). Recrystallization mechanism of as-cast AZ91 magnesium alloy during hot compressive deformation. Mater. Sci. Eng. A.

[19] Al-Samman T., Molodov K.D., Molodov D.A., Gottstein G., Suwas S. (2013). Softening and dynamic recrystallization in magnesium single crystals during c-axis compression. Acta Mater..

[20] Guo F., Zhang D.F., Fan X.W., Jiang L.Y., Yu D.L., Pan F.S. (2016). Deformation behavior of AZ31 Mg alloys sheet during large strain hot rolling process: A study on microstructure and texture evolutions of an intermediate-rolled sheet. J. Alloy. Comp..

[21] Guo F., Zhang D.F., Wu H.Y., Jiang L.Y., Pan F.S. (2017). The role of Al content on deformation behavior and related texture evolution during hot rolling of Mg–Al–Zn alloys. J Alloy. Comp..

[22] Su J., Sanjari M., Kabir A.S.H., Jung I.H., Jonas J.J., Yue S., Utsunomiya H. (2015). Characteristics of magnesium AZ31 alloys subjected to high speed rolling. Mater. Sci. Eng. A.

[23] Anbuselvan S., Ramanathan S. (2010). Hot deformation and processing maps of extruded ZE41A magnesium alloy. Mater. Des..

[24] Kliber J., Aksenov S., Fabik R. (2009). Numerical study of deformation characteristics in plane strain compression test (PSCT) volume certified following microstructure. Metalurgija.

